# Antibiotic-induced dysbiosis in the SCIME™ recapitulates microbial community diversity and metabolites modulation of *in vivo* disease

**DOI:** 10.3389/fmicb.2024.1455839

**Published:** 2024-09-12

**Authors:** Elena Dalle Vedove, Alessia Benvenga, Gianluca Nicolai, Marcella Massimini, Maria Veronica Giordano, Francesco Di Pierro, Benedetta Bachetti

**Affiliations:** ^1^R&D Division, C.I.A.M. Srl, Ascoli Piceno, Italy; ^2^Department of Veterinary Medicine, University of Teramo, Teramo, Italy; ^3^Endovet Professional Association, Roseto degli Abruzzi, Italy; ^4^Department of Medicine and Surgery, University of Insubria, Varese, Italy; ^5^Scientific and Research Department, Velleja Research, Milan, Italy

**Keywords:** gut disease, dog, intestinal microbiota, *in vitro* alternative to animal testing, prebiotics

## Abstract

**Establishing the context:**

Intestinal dysbiosis is a significant concern among dog owners, and the gut health of pets is an emerging research field. In this context, the Simulator of the Canine Intestinal Microbial Ecosystem (SCIME™) was recently developed and validated with *in vivo* data.

**Stating the purpose/introducing the study:**

The current study presents a further application of this model by using amoxicillin and clavulanic acid to induce dysbiosis, aiming to provoke changes in microbial community and metabolite production, which are well-known markers of the disease *in vivo*.

**Describing methodology:**

Following the induction of dysbiosis, prebiotic supplementation was tested to investigate the potential for microbiota recovery under different dietary conditions.

**Presenting the results:**

The results showed that antibiotic stimulation in the SCIME™ model can produce significant changes in microbial communities and metabolic activity, including a decrease in microbial richness, a reduction in propionic acid production, and alterations in microbial composition. Additionally, changes in ammonium and butyric acid levels induced by the tested diets were observed.

**Discussing the findings:**

This alteration in microbial community and metabolites production mimicks *in vivo* canine dysbiosis patterns. A novel dynamic *in vitro* model simulating canine antibiotic-induced dysbiosis, capable of reproducing microbial and metabolic changes observed *in vivo*, has been developed and is suitable for testing the effects of nutritional changes.

## 1 Introduction

Gastrointestinal diseases are one of the primary reasons why dog owners in Western countries visit veterinarians (Hubbard et al., 2007; Nationwide Mutual Insurance Company, 2023). Similar to humans, intestinal disorders in dogs often correlate with an alteration in the intestinal microbiota, termed dysbiosis (Guard et al., 2015; Suchodolski, 2016). Dysbiosis in the gut can manifest as changes in microbial composition (e.g., microbial richness, bacteria ratio) and alterations in metabolites production (e.g., decreased synthesis of short-chain fatty acids or increased production of putrefaction markers, such as ammonia; Suchodolski, 2022). The modulation of the intestinal microbial community and its metabolic function is of growing interest, and new *in vitro* models are valuable for advancing knowledge in this area without necessitating animal testing. Recently, within veterinary medicine, considerable attention has been directed toward strategies aimed at modulating the composition and metabolism of the canine intestinal microbial population as a potential new approach to enhancing canine health (Pinna and Biagi, 2014).

In this context, the Simulator of the Canine Intestinal Microbial Ecosystem (SCIME™), was developed and validated as an alternative to *in vivo* trials (Duysburgh et al., 2020). SCIME™ is a semi-dynamic *in vitro* model designed to simulate the canine gastrointestinal tract, focusing on the intestinal microbiota (Duysburgh et al., 2020). A standard SCIME™ setup consists of reactors that simulate the stomach, small intestine, and proximal (PC) and distal colon (DC) of dogs (Duysburgh et al., 2020). Once stabilization of colonic microbiota occurs, the simulated canine microbial community composition closely resembles the *in vivo* situation (Duysburgh et al., 2020). Moreover, a primary advantage of the simulator, compared to *in vivo* studies, is that by strictly controlling the environmental factors, it can provide mechanistic insights on how treatments work (Duysburgh et al., 2021).

In addition to the study of healthy microbiota, *in vitro* models can be useful to reproduce pathologic conditions, such as dysbiosis. Currently, the SCIME™ has been exclusively utilized for simulating intestinal microbiota in healthy conditions, whereas its human counterpart, the SHIME^®^ (the Simulator of the Human Intestinal Microbial Ecosystem) has been adapted and is already extensively employed to mimic intestinal dysbiosis (Ichim et al., 2018; El Hage et al., 2019; Marzorati et al., 2020; Duysburgh et al., 2021). In the present study, broad-spectrum antibiotics were used to induce microbial dysbiosis, as previously reported in the literature, where experiments were conducted using the SHIME^®^ model (Marzorati et al., 2017, 2020; Ichim et al., 2018; El Hage et al., 2019; Duysburgh et al., 2021). Amoxicillin-clavulanic acid was selected owing to its frequent usage as an antimicrobial agents in gastrointestinal disease in dogs and cats, and its association with gastro-intestinal disorders and antibiotic-associated diarrhea, which are reported as side effects (German et al., 2010; Jones et al., 2014; Mancabelli et al., 2021; Zoetis UK Limited, 2024). This study aims to investigate the feasibility of *in vitro* replication of a condition that mimics, in terms of taxonomic and biodiversity characteristics, the dysbiosis observed in dogs under various circumstances, including antibiotic administration (regardless of diarrheal symptoms) and other instances such as episodes of diarrhea due to gastroenteritis, functional gastrointestinal disorders, or Inflammatory Bowel Disease (IBD).

The primary objective of the current study was to investigate whether the administration of a specific dose of Amoxicillin-clavulanic acid to a dog with a healthy canine microbiota (eubiosis) could determine dysbiosis and induce alterations in metabolites production, the same markers indicative of dysbiosis *in vivo*. Moreover, industrial diets often lack essential nutrients such as fibers and several studies have shown the potential efficacy of prebiotics in nutrition, especially in mitigating the deleterious effect of antimicrobial treatments on the intestinal microbiota by facilitating faster restoration of gut homeostasis through eubiosis (Sanders et al., 2019). For this reason, an ancillary objective of the experiment was to assess the potential of the microbiota recovery under different dietary conditions. To achieve these goals, the SCIME™ was utilized coupled with 16S-targeted Illumina sequencing and metabolomics analysis.

## 2 Materials and methods

### 2.1 Fecal sample collection

Fecal samples for SCIME™ inoculum were collected in closed containers in the presence of an Oxoid™ AnaeroGen™ bag (Oxoid, Basingstoke, Great Britain), to remove all oxygen from the environment, and stored at 4°C until further processing. Samples were homogenized in anaerobic phosphate buffer, containing 8.8 g/L K_2_HPO_4_, 6.8 g/L KH_2_PO_4_, 0.1 g/L sodium thioglycolate, and 0.015 g/L sodium dithionite (20% w/v), and fecal supernatant was collected upon centrifugation and immediately used. As shown in Supplementary Table 1, to account for biological variability, fecal samples of six different clinical healthy adult dog donors were used to inoculate the distal colonic compartments of the SCIME™. Dog breeds involved in the experiment included Toy Poodle, Springer Spaniel, Cane Corso, Labrador Retriever, Jack Russell Terrier and a mixed-breed dog. All dogs were privately owned, lived in various home environments, and were fed various commercial diets. None of the dogs had a history of gastrointestinal signs or received antibiotics for at least 6 months prior to fecal samples collection. All healthy dogs lived in Ascoli Piceno, Italy. All dogs were neutered female; four were adult and two mature. All animal procedures were carried out in accordance with national guidelines.

### 2.2 Simulator of the canine microbial ecosystem

**Figure 1:**
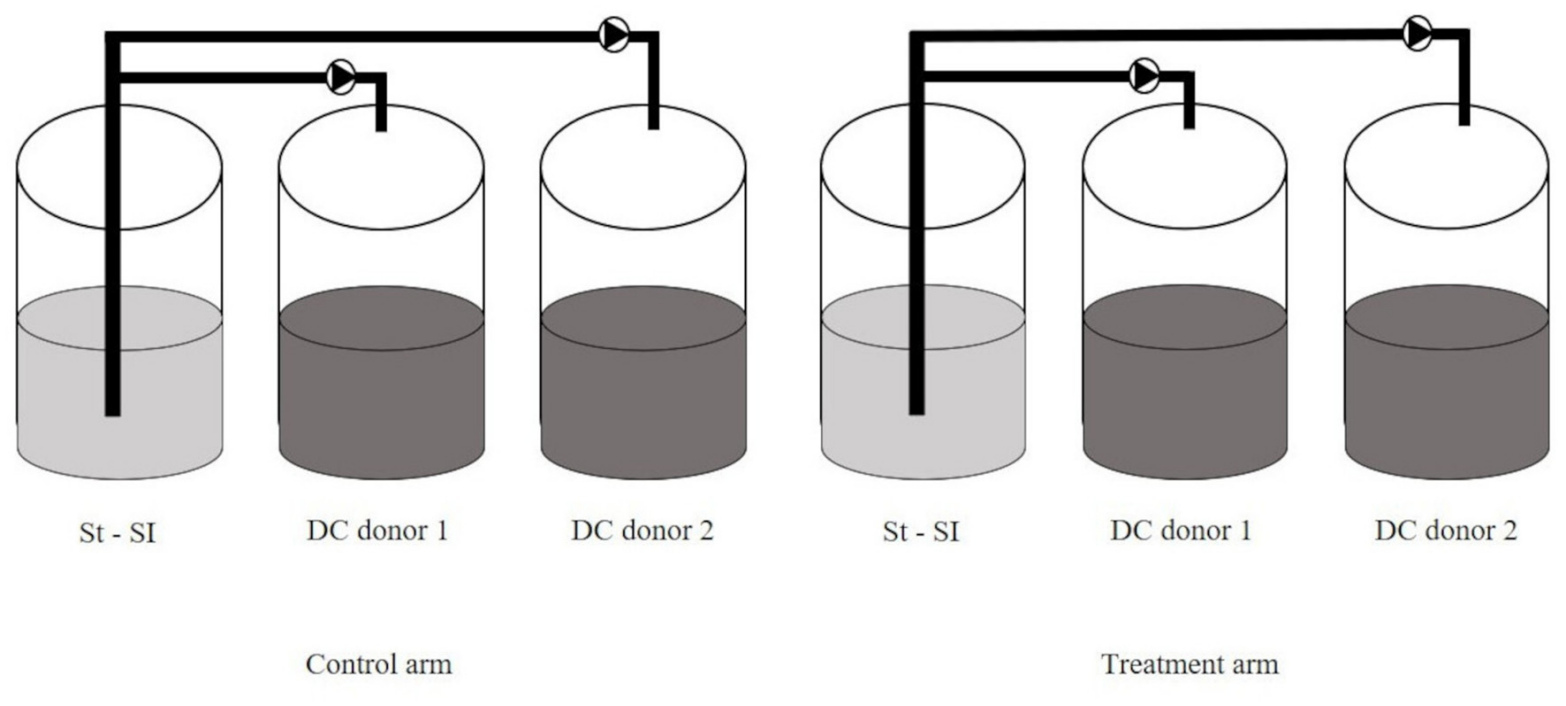
SCIME™ reactor setup. Schematic overview of the Simulator of the Canine Intestinal Microbial Ecosystem (SCIME™) simulating the canine gastrointestinal tract (St, Stomach; SI, Small Intestine; DC, Distal Colon). Two donors were used per run and three runs were performed.

The configuration of the SCIME™ reactor was adapted from the SHIME^®^ model (ProDigest, Ghent, Belgium and Ghent University, Ghent, Belgium) as previously described by Duysburgh et al. (2020) and Verstrepen et al. (2021). The set-up used in this study consisted of a stomach/small intestine (St-SI) vessel and two distal colon (DC) vessels (non-treated arm and treated arm) for two canine donors in parallel, per run. Three runs including 2 donors/run were performed, to account for biological variability. In this trial, proximal colon (PC) vessel was not used because the number of donors per run was prioritized over the number of colons, as it was done in previous experiment using the SHIME^®^ (El Hage et al., 2019). The selection of the DC vessel over the PC vessel was based on its characterization by a microbial community abundant in species with specific metabolic functions, such as protein degradation, therefore more interesting outputs were expected (Duysburgh et al., 2020). The experimental setup for one run is shown in Figure 1.

Briefly, for each run, the set-up consisted of two pairs of three double-jacketed vessels connected via peristaltic pumps and operated under strictly anaerobic condition. These vessels simulate the stomach and small intestine (St-Si, simulated in one compartment by modifying conditions over time). For the reasons mentioned above, the colon vessels were limited to the distal colon (DC, pH 6.5–6.9). To simulate the gut microbiome, the distal colon vessels were inoculated with microbiota isolated from a fecal sample originating from healthy dogs with no history of antibiotic treatment in the 6 months prior to sample collection, as previously described.

The first vessel (St-SI) was fed with a nutritional medium (indicated as SCIME™ “Feed”) prepared by dissolving commercial dog petfood (composition shown in Table 1) at 9 g/L in gastric juice [1.5 g/L yeast extract, 4 g/L special peptone (Oxoid), 4 g/L mucin, and 0.5 g/L L-cystein (Sigma-Aldrich)].

**Table 1:** Dog feeds composition and analytical components.

**Standard petfood**	**Prebiotic-enriched petfood**
**Composition:** Dehydrated chicken protein (28%), rice (28%), maize (26%), chicken fat (7%), dehydrated fish protein, dried beet pulp (4%), fish oil (2%), sodium chloride, dried brewer's yeast (0.3%).	**Composition:** Derivatives of vegetable origin [of which dried beet pulp (4%), cellulose (2.5%), dried chicory (0.5%), yucca (0.1%)], processed chicken proteins (19%), animal fat, dried gelatine (1.25%), brewer's yeast [of which, mannan-oligosaccharides (MOS; 0.5%), beta-glucans (0.5%)], hydrolysed collagen (0.75%), dried apple pulp.
**Additives per kg:** Nutritional additives: Vitamin A 10,000 IU; Vitamin D3 1,000 IU; Vitamin E 100 mg; Vitamin C 100 mg; Niacin 25 mg; Calcium D-pantothenate 10 mg; Vitamin B2 5 mg; Vitamin B6 4 mg; Vitamin B1 3 mg; Biotin 0.25 mg; Folic acid 0.30 mg; Vitamin B12 0.04 mg; Choline chloride 1,500 mg; Zinc (zinc oxide): 86.7 mg; Zinc (zinc sulfate monohydrate): 43.7 mg; Manganese (manganous sulfate monohydrate): 48.8 mg; Iron [iron (II) sulfate monohydrate]: 14.5 mg; Iron [iron (II) carbonate]: 28.9 mg; Copper [copper (II) sulfate pentahydrate]: 12.8 mg; Iodine (anhydrous calcium iodate): 1.56 mg; Selenium (sodium selenite): 0.101 mg; DL-Methionine, technically pure 1,500 mg.	**Additives per kg:** Technological additives: antioxidants, preservatives—Organoleptic additives: tannic acid (410 mg).
**Analytical components:** crude protein 25.00%; crude fat 12.00%; crude fibers 2.00%; raw ash 6.50%; Calcium 1.20%; Phosphorus 0.90%.	**Analytical components:** crude protein: 16.50%, crude fat: 3.50%, crude fiber: 3.50%, raw ash: 2.50%

During weeks 1–5 both non-treated arm and treated arm were given standard petfood, included in SCIME™ Feed. During week 6, non-treated arm was still fed with standard petfood, while treated arm was switched to prebiotic-enriched petfood.

**Figure 2:**
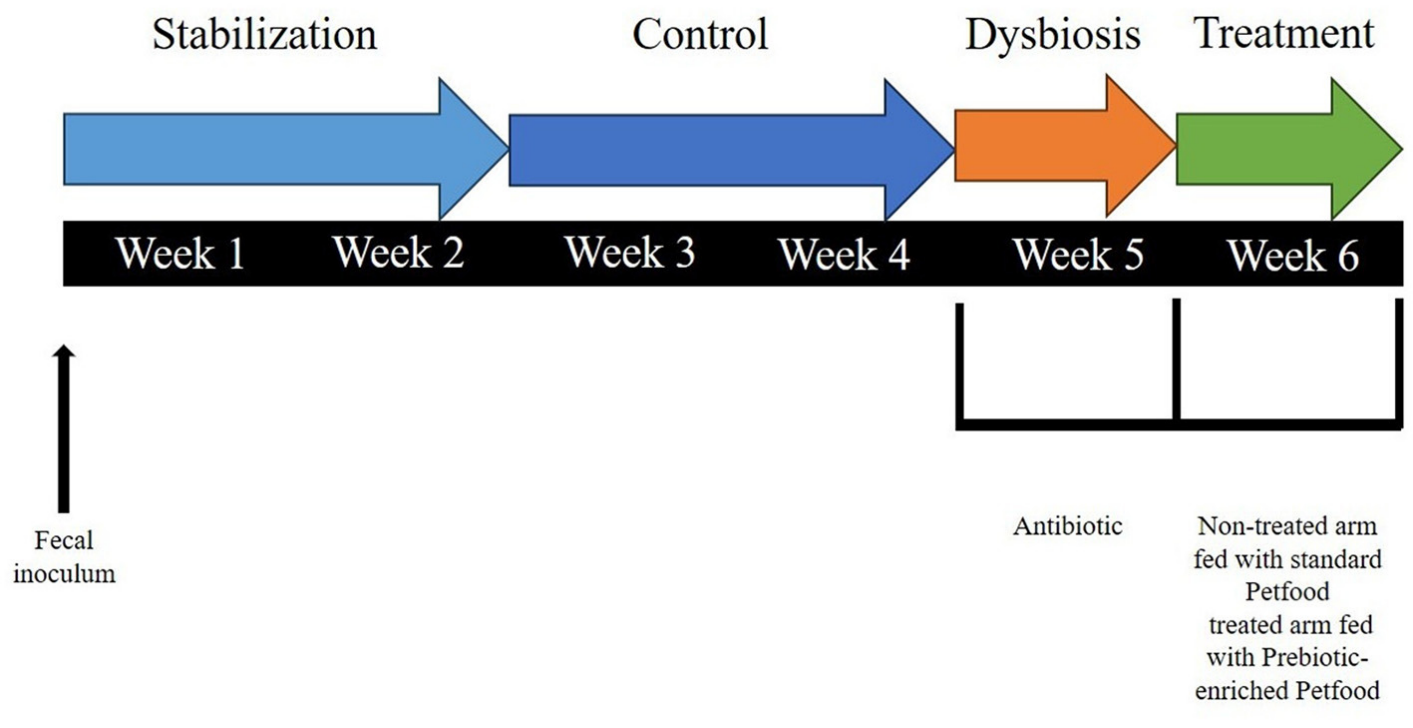
Schematic representation of the experimental setup for each run, including the experimental periods, i.e., stabilization period (2 weeks), control period (CTRL; 2 weeks), dysbiosis period (AB; 3 days), and treatment period (TR; 1 week).

The commercial diet was previously grinded and, after homogenization, the feed was autoclaved, mixed and decanted after 10′ of sedimentation. Twice daily, the St-SI reactor was filled with 140 mL Feed and 60 mL pancreatic juice [12.5 g/L NaHCO_3_, 2 g/L oxgall (Difco), and 0.9. g/L pancreatin (Applichem)], resulting in a final concentration of 6.3 g of dog feed/L. The colon reactors were continuously stirred with constant volume (DC: 167 mL) and pH control. The pH controllers, peristaltic pumps for liquid transfer and flushing equipment were incorporated in an automated setup controlled by LabVIEW software (SHIME^®^, ProDigest). The system was run at 39°C under anaerobic conditions with daily flushing with nitrogen gas. The experimental schedule is schematically shown in Figure 2.

There was a 2-week stabilization period to allow the microbiota to adapt to the *in vitro* environment, followed by a 2-week control period during which stability in the microbiome was established and baseline parameters were measured. At the completion of the control period, there was a 1-week pre-treatment period (Dysbiosis week). Amoxicillin: Potassium Clavulanate (2:1; TOKU-E; 45 ppm, twice daily) was added to each colon vessel for 3 days to induce dysbiosis of the microbiota. This antibiotic dosage was determined based on previous experiments run with SHIME^®^ (Duysburgh et al., 2021) and considering both the daily dosage recommended for dogs (Zoetis UK Limited, 2024) and the absorption rate (Kung and Wanner, 1994; The European Agency for the Evaluation of Medicinal Products, 1996). During week 1–5, both the non-treated arm and treated arm were given Feed including standard petfood. During week 6, the non-treated arm continued to be fed with Feed including standard petfood (non-treated group), while the treated arm was switched to Feed with prebiotic-enriched petfood instead (treated group). The composition of the two commercial diets used in the experiment are shown in Table 1.

### 2.3 Sample collection

Sampling of each distal colon vessel was performed three times per week during the stabilization period, control period, dysbiosis and treatment period. Specifically, sampling was conducted on Monday, Wednesday, Friday during stabilization, control and treatment period, while during the dysbiosis week, sampling occurred on Tuesday, Wednesday, and Thursday, which were the 3 days of the antibiotic treatment. Liquid samples for subsequent analysis of microbial metabolic activity were immediately frozen at −20°C, while pelleted cells (5 min, 9,000 g) originating from 1 mL liquid sample were frozen at −20°C for subsequent molecular analysis.

### 2.4 Microbial metabolic activity

The parameters used to assess the activity of the gut microbiota in the colons were monitored three times per week from the stabilization period onwards. Levels of short-chain fatty acids (SCFAs, acetate, propionate and butyrate) and branched-chain fatty acids (BCFAs, isobutyrate, isovalerate, and isocaproate) were quantified with gas chromatography (GC) coupled to flame ionization detection (FID). After the addition of 2-methyl hexanoic acid as an internal standard, 2.0 mL of sample was extracted with diethyl ether. The extracts were analysed using an Agilent 7890B GC gas chromatograph (Agilent, Santa Clara, CA, United States), equipped with a GC DB-FATWAX Ultra inert capillary column (length: 30 m; Inner diameter: 0.32 mm; Film thickness 0.25 μm, Agilent, Santa Clara, CA, United States), a flame ionization detector and a split injector. The injection volume was 1 μL and the column temperature profile was set from 110 to 160°C, with a temperature increase of 6°C min−1. Helium was used as the carrier gas and the injector and detector temperatures of the were both 200°C. The procedure was adapted from what previously described by Ghyselinck et al. (2020).

Ammonium analysis was performed by steam distillation adapted from what was previously described by De Wiele et al. (2004). Using a Kjelmaster K-375 (BÜCHI, Flawil, Swizerland), ammonium in the sample was liberated as ammonia by the distillation in an alkalin medium (by addition of 32% NaOH). The released ammonia was captured from the sample into a boric acid mixed indicator solution, creating an ammonium-borate complex. The ammonium in the distillate was determined by titration with HCl.

### 2.5 Microbial community composition

The microbial community composition was determined through Illumina sequencing (16S rRNA) and performed by an external laboratory (Genprobio, Cadorago, Italy). Frozen samples from the three runs were shipped to the laboratory under frozen conditions where they were preserved at −20°C, until processed. Next generation 16S rRNA gene amplicon sequencing of V3 region was performed, using the primers 341F (CCATCTCATCCCTGCGTGTCTCCGAC) and 519R (CCTCTCTATGGGCAGTCGGTGAT), with the procedure described by Milani et al. (2013). Results were delivered in the form of relative abundances for each sample to the level of genera, prediction of relative abundances in term of species and alpha diversity curves. Since also raw data was delivered, other analyses were run using QIIME2 (Bolyen et al., 2019).

### 2.6 Statistical analysis

Statistical analysis was performed through R software (3.6.3, 2020) and Excel [Microsoft Corporation. (2018). Microsoft Excel. Retrieved from https://office.microsoft.com/excel].

To test the significance of metabolites and relative bacteria abundances, and to account for the correlation between repeated measurements on the same subject, a mixed-effect model was used, which considered as fixed effect the following groups: control period (CTR), antibiotic stimulation (AB), and non-treated arm (NT) and treated arm (TR) during the treatment period. The donor was considered as random effect. To test the differences among groups, the Tukey contrasts *post-hoc* test was used.

## 3 Results

### 3.1 Microbial community composition

**Figure 3:**
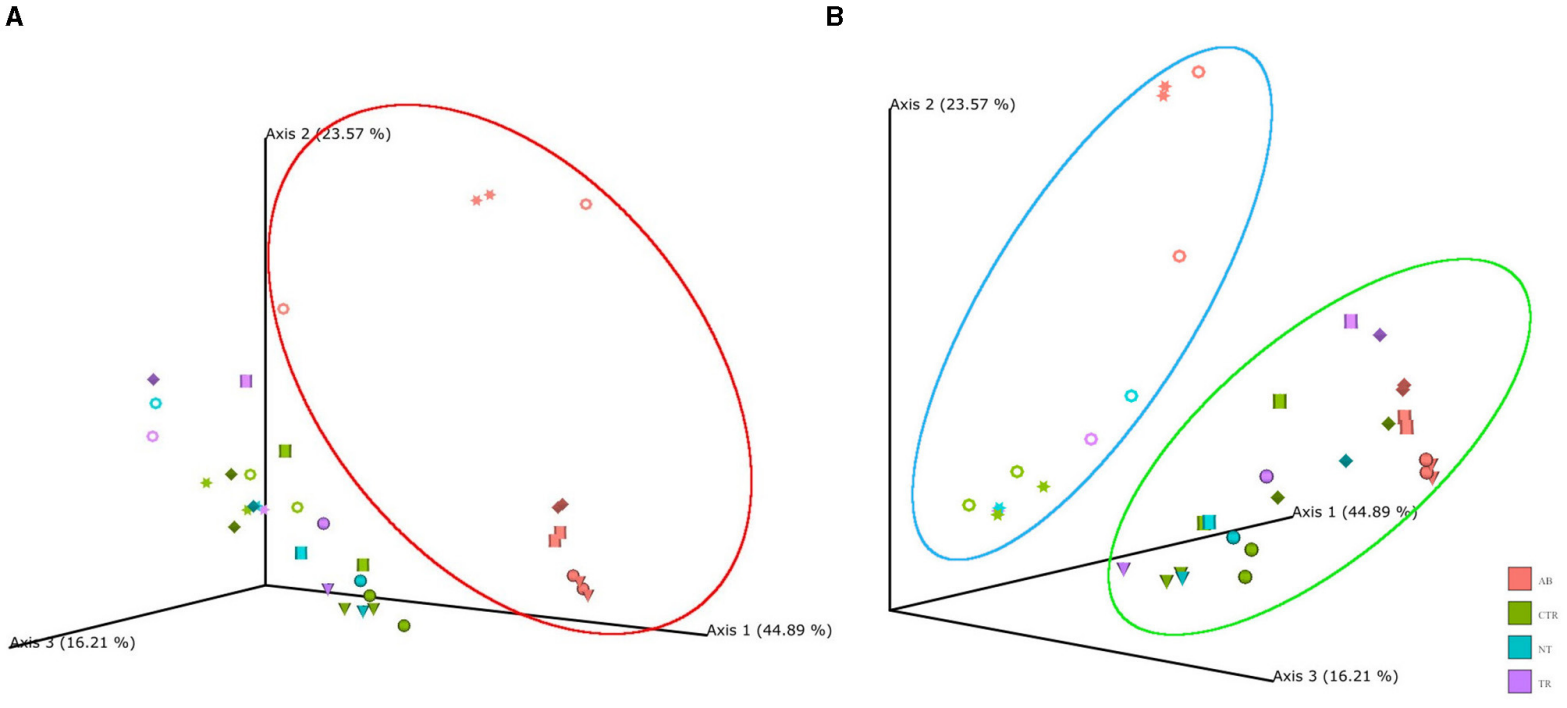
Principal coordinate analysis (PCoA) plot based on weighted UniFrac distances. PCoA was used to plot beta diversity of SCIME™ samples. The colors are associated with the group (AB, antibiotics; CTR, control; NT, non-treated arm; TR, treated arm), while the shapes are associated with the donors (Ring = D1, Cylinder = D2, Sphere = D3, Cone = D4, Star = D5, Diamond = D6). **(A)** Shows that AB group clustered in red circle. **(B)** Shows that in the blue circle there are donors 1 and 5, while the rest are clustered in the green circle.

In beta diversity PCoA plots (Figure 3A) based on weighted UniFrac analysis, the samples associated with antibiotic stimulation (AB) are distinctly clustered apart from the other groups. It is noteworthy that PCoA based on other metrics (such as Jaccard, Bray-Curtis, and unweighted UniFrac) show the same AB cloud of samples separated from the rest also depict a distinct cluster of antibiotic-stimulated samples separated from the rest (data not shown). PCoA plots were constructed to compare the groups across the weeks and revealing that samples related to the antibiotic stimulation can be identified as a cluster (red contour, Figure 3A) separated from the other samples. This represents healthy microbiota before antibiotic stimulation and bacterial communities one week after its end, both in the non-treated arm (standard petfood) and the treated arm (prebiotic-enriched petfood). Samples from control week and one week after the end of antibiotic stimulation overlap. Moreover, two additional clusters can be identified, both in PCoA plots based on weighted UniFrac metrics; in Figure 3B samples belonging to donors 1 and 5 (identified with circle and star shapes) and to donors 2, 3, 4, and 6 clustered separately. Furthermore, the PCoA plots show that all samples taken during dysbiosis week (AB) tend to move to the same direction in the graph regardless of the diversity of donors and microbiota.

PERMANOVA was used to determine factors that explained variance in bacterial community. The input of PERMANOVA was the weighted UniFrac distance matrix of 16s rRNA data and, the test was run in qiime2 environment. This test indicates that the differences among the groups were statistically significant (*p*-value = 0.001, num. of permutations = 999). The subsequent pairwise test shows that the differences between AB group and the others were statistically significant (AB vs. CTR *p*-value = 0.001; AB vs. NT *p*-value = 0.001; AB vs. TR *p*-value = 0.001). To test whether significant PERMANOVA results were based on location or dispersion effects, the PERMDISP routine was applied to evaluate the homogeneity of multivariate dispersions among groups. Since PERMDISP test did not show any significant difference (*p*-value = 0.64, num. of permutations = 999), it can be assessed that the significant differences highlighted by the PERMANOVA test cannot be ascribed to the variance within group, but to the antibiotic effect.

**Figure 4:**
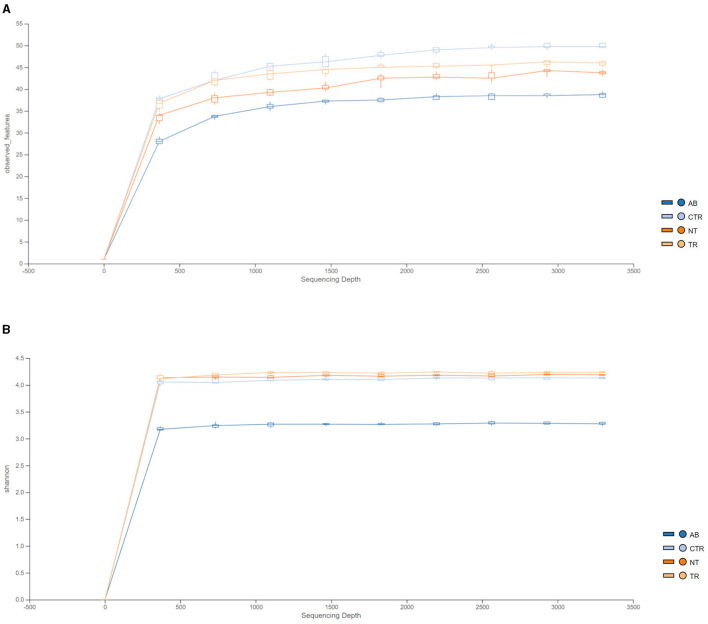
Rarefaction curves of 16 S rRNA gene sequences, expressed as OTUs **(A)** and Shannon index **(B)** separated by groups. Different colors represent different groups (AB = antibiotic stimulation, CTR = control period, NT = non-treated microbiota, TR = treated microbiota).

Figure 4 illustrates the rarefaction curves for observed species (represented by OTUs, operational taxonomic units) and Shannon index.

Figure 4A shows that observed species decreased in the dysbiosis week (AB) compared to the control week (CTR). Furthermore, one week after the end of antibiotic stimulation, the number of observed species had increased both in the non-treated arm and in the treated arm compared to the dysbiosis period. In addition, Figure 4A shows that OTUs in the treated arm were greater than in the non-treated arm, but the curve is still lower compared to the previous healthy microbiota (control period).

**Figure 5:**
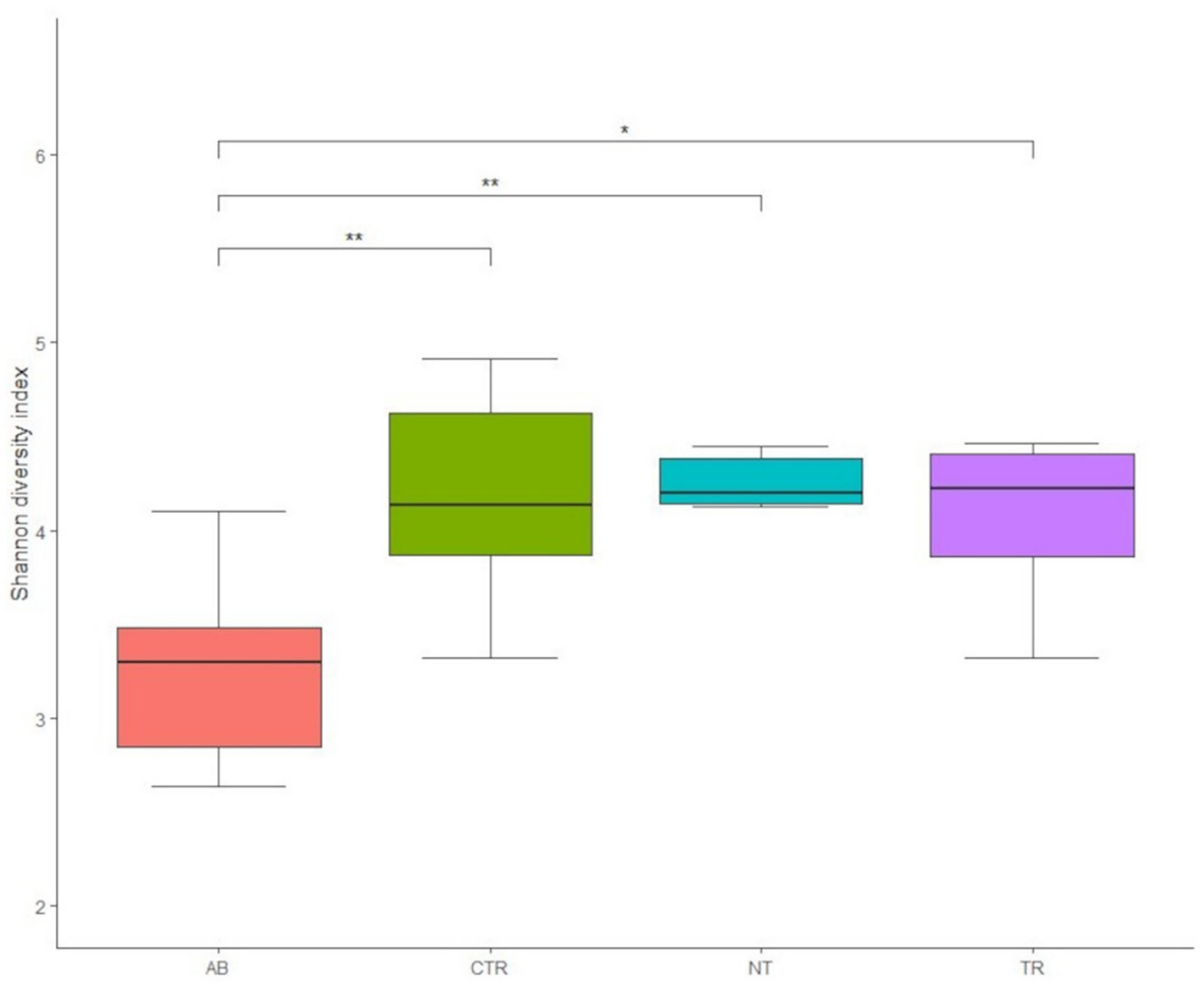
Boxplots of Shannon diversity index values of groups (AB = antibiotic stimulation, CTR = control period, NT = non-treated microbiota, TR = treated microbiota). There are significant differences (marked with asterisks) between all weeks when compared to the antibiotic week; the lowest value of the index is found in the antibiotic week.

Figure 4B displays the rarefaction curves for the Shannon index. The curve for dysbiosis week (AB) is significantly decreased compared to the curve for control week and to the curves for treated and non-treated microbiota. The Shannon index was used to assess species richness. As shown in Figure 4B, lower values of the index were observed in the antibiotic stimulation group, as expected. Indeed, a Kruskal-Wallis test, performed on all groups, revealed a significant difference (*p*-value = 0.006); further differences among groups were elucidated with a *post-hoc* test (pairwise *t*-test; Figure 5). There are significant differences in all groups when compared to the antibiotic stimulation (AB vs. CTR *p*-value = 0.003; AB vs. NT *p*-value = 0.009; AB vs. TR *p*-value = 0.019). OTU and Chao1 were also evaluated as alpha diversity indexes, they behaved similarly to Shannon index (Supplementary Table 2).

**Figure 6:**
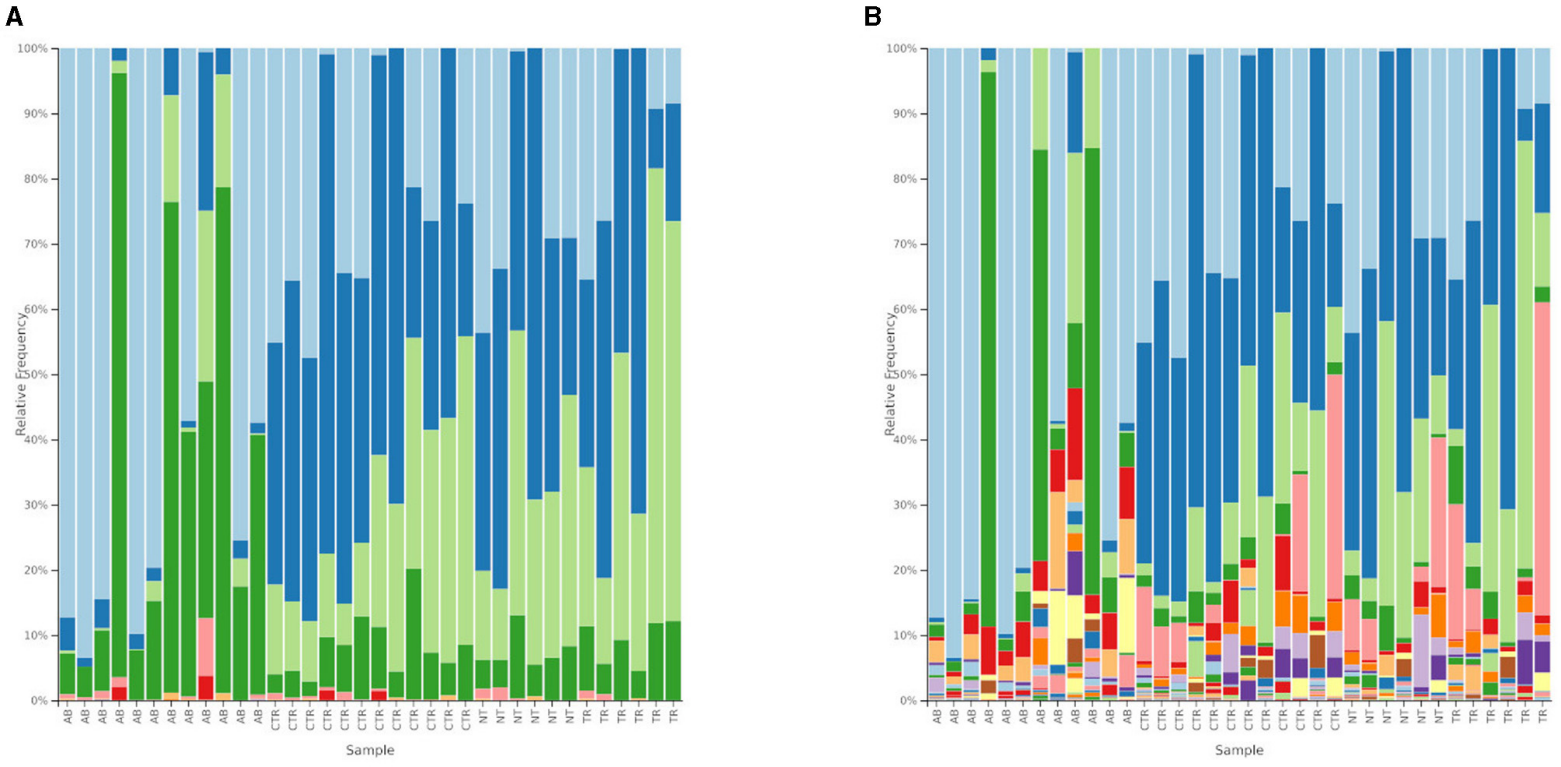
Relative abundances of bacterial composition. **(A)** Shows bacterial composition at phylum level. Light blue = Fusobacteria; blue = Bacillota; light green = Bacteroidota; green = Pseudomonadota; pink = Actinomycetota; red = Synergistes; light orange = Desulfobacterota. The groups are reported on the x-axis named as AB = antibiotic stimulation, CTR = control period, NT = non-treated microbiota, TR = treated microbiota. **(B)** Shows bacterial composition at genus level. Legend of the 10 most abundant genus: light blue = *Fusobacterium*; blue = *Megamonas*; light green = *Bacteroides*; green = *Escherichia-Shigella*; pink = *Prevotella*; red = *Pseudomonas*; light orange = *Parasutterella*; orange = *Alloprevotella*; light purple = *Phascolarctobacterium*; purple = *Sutterella*; light yellow = *Delftia*. The groups are reported on the x-axis named as AB = antibiotic stimulation, CTR = control period, NT = non-treated microbiota, TR = treated microbiota.

**Figure 7:**
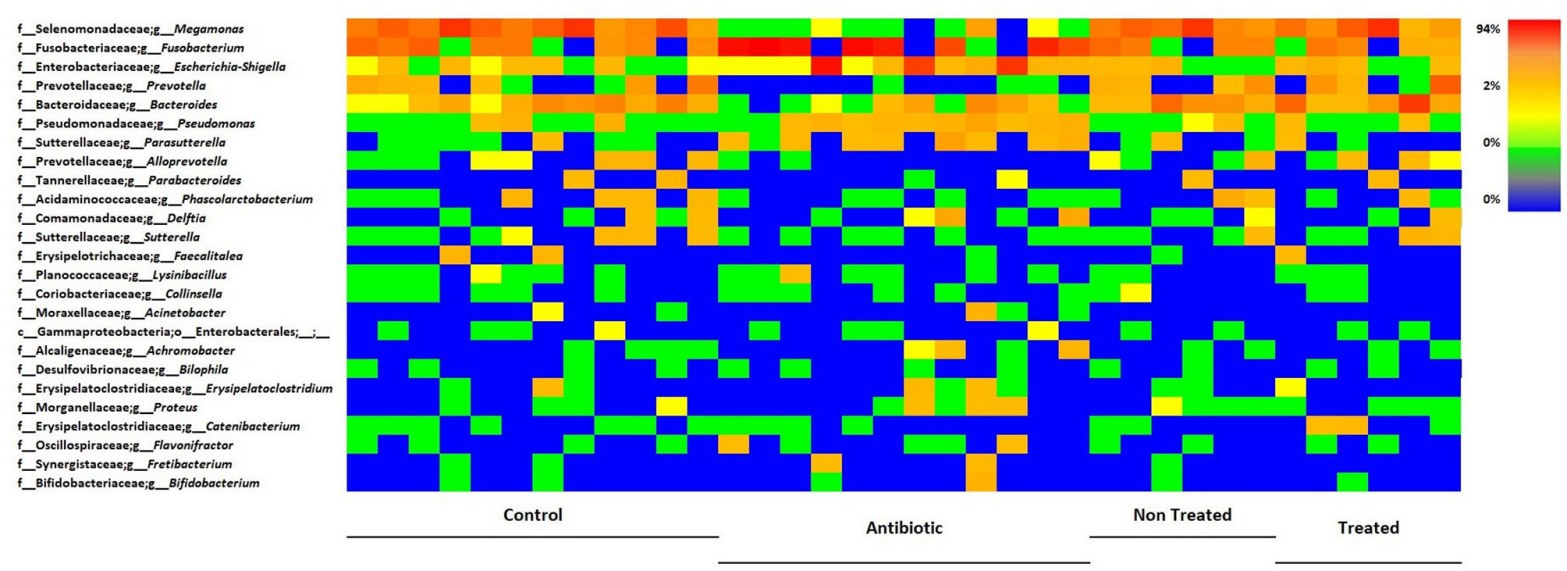
Heatmap illustrating the relative abundance of predominant bacterial genera in samples, divided by group. Each line reports the changes in relative abundance of a bacterial genus, among experimental groups, each column represents the bacteria relative abundances of a sample.

Figures 6, 7 and Tables 2, 3 summarize differences in bacterial groups between groups. The groups are control (CTR), antibiotic stimulation (AB), prebiotic-enriched feed, labeled “treated” (TR), and normal feed, labeled “non-treated” (NT). Sequences belonging to the phyla Bacillota and Bacteroidota were significantly decreased after antibiotic stimulation, compared to control weeks (*p* < 0.001 and < 0.001, respectively). Conversely, sequences belonging to the phyla Fusobacteria and Pseudomonadota were significantly increased after antibiotic trigger, compared to control weeks (*p* < 0.001 and < 0.001, respectively). Moreover, sequences belonging to the genus *Megamonas* and *Alloprevotella* significantly decreased after antibiotic stimulation, compared to control weeks (*p* < 0.001 and 0.0545, respectively). Conversely, sequences belonging to the genus *Fusobacterium, Shigella, Pseudomonas*, and *Parasutterella* were significantly increased after antibiotic trigger, compared to the control weeks (*p* < 0.001 = 0.0426, 0.00910, 0.0395, respectively).

**Table 2:** Percentages of the most abundant bacterial groups.

**Phylum**	**CTR**	**AB**	**NT**	**TR**
Bacillota	44.93 (20.34–76.62)	2.61 (1.02–24.4)	40.87 (24.13–69.24)	37.72 (9.22–71.42)
Fusobacteria	25.16 (0–47.47)	66.42 (0.00–93.5)	29.09 (0.00–43.67)	8.84 (0–35.50)
Pseudomonadota	7.23 (2.27–20.07)	26.91 (4.70–92.6)	5.70 (4.25–12.79)	9.59 (4.18–12.15)
Bacteroidota	19.7 (6.34–47.33)	1.23 (0–26.12)	25.37 (10.83–43.62)	34.15 (13.09–69.69)
Actinomycetota	0.21 (0–1.25)	0.37 (0.00–8.79)	0.12 (0.00–1.97)	0.00 (0–1.22)
Desulfobacterota	0 (0–0.64)	0 (0.00–1.08)	0 (0.00–0.61)	0 (0.00–0.26)
Synergistes	0 (0–1.49)	0 (0.00–3.69)	0 (0.00–0.04)	0 (0.00–0.00)

Median (min-max) in percent is shown. The groups are reported named as (AB = antibiotic stimulation, CTR = control period, NT = non-treated microbiota, TR = treated microbiota). The abbreviation “unclass.” denotes an unclassified taxonomy within the respective taxonomic group.

**Table 3:** *P*-values of the most abundant bacteria at phylum and genus level, obtained from multiple comparisons of means (Tukey contrasts) *post-hoc* test.

**Comparison**						
**Phylum**	**CTR-AB**	**AB-NTR**	**AB-TR**	**CTR-NTR**	**CTR-TR**	**NTR-TR**
Bacillota	< 0.001	< 0.001	< 0.001			
Fusobacteria	< 0.001	< 0.001	< 0.001			
Pseudomonadota	< 0.001	0.00499	0.00987			
Bacteroidota	< 0.001	0.00487	0.01019			
Actinomycetota						
Desulfobacteria						
Synergistota						
Unclass. bacteria						
Unassigned						
Verrucomicrobiota						
Unassigned						
**Genus**	**CTR-AB**	**AB-NTR**	**AB-TR**	**CTR-NTR**	**CTR-TR**	**NTR-TR**
Megamonas	< 0.001	< 0.001	< 0.001			
Fusobacterium	< 0.001	< 0.001	< 0.001			
Shigella	0.0426					
Prevotella			0.00364			
Bacteroides			0.00163			
Pseudomonas	0.00910	0.00635	0.00486			
Parasutterella	0.0395	0.04430	0.02529			
Alloprevotella	0.0545	0.04119				
Parabacteroides						
Phascolarctobacterium		0.0203				
Delftia						
Sutterella			0.00795			
Faecalitalea						
Acinetobacter						
Lysinibacillus			0.0458			
Collinsella						
Enterobacterales						
Achromobacter	0.0379					
Bilophila						
Erysipelatoclostridium						
Proteus						
Catenibacterium			0.00317		0.00862	
Flavonifractor	0.0389					
Fretibacterium						

The groups are reported named as AB = antibiotic stimulation, CTR = control period, NT = non-treated microbiota, and TR = treated microbiota.

One week after the end of antibiotic stimulation, no significant differences were identified between samples from treated or non-treated arm when evaluating bacterial groups. However, sequences belonging to the phyla Bacillota (*p* < 0.001 both TR and NT) and Bacteroidota (*p* = 0.01019 and *p* = 0.00487, TR and NT, respectively) were significantly increased after antibiotic stimulation compared to the previous week, both in treated and non-treated arms. Conversely, sequences belonging to the phyla Fusobacteria (*p* < 1e^−04^ both TR and NT) and Pseudomonadota (*p* = 0.00987 and *p* = 0.00499, TR and NT, respectively) were both significantly decreased after antibiotic stimulation compared to the previous antibiotic week, both in treated and untreated arms. At genus level, *Megamonas* increased in both treated and non-treated arm (*p* < 0.001, both), while *Pseudomonas* (*p* = 0.00486 and *p* = 0.00635 TR and NT, respectively) and *Parasutterella* (*p* = 0.02529 and *p* = 0.04430 TR and NT, respectively) decreased in in both treated and non-treated arm. Some differences in microbial communities at genus level can be seen comparing samples taken during antibiotic week to those ones taken during the following week. For example, sequences belonging to genus *Prevotella* (*p* = 0.00364) and *Bacteroides* (*p* = 0.00163) significantly increased in treated arm compared to the previous antibiotic week. Additional significant differences can be seen for *Alloprevotella, Phascolarctobacterium, Sutterella, Lysinibacillus*, and *Catenibacterium*, as shown in Table 3, despite the low relative abundance of these genera (Table 2).

### 3.2 Metabolic activity analysis

Antibiotic stimulation significantly altered SCFAs levels. Plotting the most important SCFAs (Supplementary Figure 1) produced by the intestinal microbiota, a decrease of metabolites concentration is observed following the antibiotic stimulation. The major changes were observed for acetic acid, propionic acid and BCFAs, but a substantial reduction in butyric acid levels was also evident. The effect of the antibiotic, in all donors, on the production of metabolites, generated different distributions compared to other groups. Metabolites concentrations expressed in percentages for SCFAs and mg/L for ammonium are reported in Table 4.

**Table 4:** Percentages of the most common microbiota metabolites, SCFAs, BCFAs, and ammonium.

**Group**	**Acetic acid (mmol/ml)**	**Propionic acid (mmol/ml)**	**Butyric acid (mmol/ml)**	**BCFAs (mmol/ml)**	**Ammonium (mg/L)**
CTR	2.11 (3.74–7.14)	11.13 (6.67–18.01)	6.03 (3.54–7.82)	2.54 (2.14–3.66)	613.38 (487.1–703.58)
AB	2.67 (0.35–6.69)	1.34 (0.2–12.7)	8.07 (0.37–22.42)	2.02 (0.04–5.8)	432.98 (234.53–739.67)
NTR	3.75 (2.71–6.14)	10.39 (5.68–17.52)	6.59 (3.31–8.17)	2.6 (1.84–3.36)	586.32 (487.1–757.71)
TR	3.56 (2.91–6.78)	10.99 (6.09–20.22)	8.7 (4.03–11.77)	2.11 (1.82–2.76)	509.65 (432.98–595.34)

Median (min-max)^*^ in percent is shown. The groups are reported labeled as AB = antibiotic stimulation, CTR = control period, NT = non-treated microbiota, and TR = treated microbiota.

Consistently, the TR and NTR groups had significantly higher Acetate and Propionate concentrations compared to the AB group (for Acetate AB-TR *p*-value = 3.22e^−05^ and AB-NTR *p*-value = 6.47e^−05^, for Propionate AB -TR *p*-value = < 1e^−06^ and AB-NTR *p*-value = < 1e^−06^). BCFAs levels were significantly higher in NTR than in AB (*p*-value < 0.001), while they were almost unaltered in the other groups.

It is important to highlight that the TR group had significantly higher butyric acid levels compared to the CTR (*p*-value = 0.00115) and NTR (*p*-value = 0.01669), indicating that the treatment with the prebiotic seems to help with a faster recovery of the butyrate production.

Similarly, the fecal ammonium concentration (Supplementary Figure 2) decreased significantly following the antibiotic stimulation (CTR-AB, *p*-value < 0.001) while the NTR and TR groups had higher ammonium concentrations than the AB group, consistently with what is expected after finishing the antibiotic administration. The TR group had lower ammonium concentrations compared to the CTR (*p*-value = 0.00105) and the NTR (*p*-value = 0.02410) groups, suggesting a significant effect of the treatment, while the ammonium concentration of NTR did not show significant differences compared to the control, even though it showed higher values. The statistical test results are summarized in Table 5.

**Table 5:** Significances of the main SCFAs concentrations obtained from multiple comparisons of means (Tukey contrasts) *post-hoc* test.

**Comparisons**						
**Metabolite**	**CTR-AB**	**AB-NT**	**AB-TR**	**CTR-NT**	**CTR-TR**	**NT-TR**
Acetic acid	^***^↓	^***^↑	^***^↑			
Butyric acid	^*^↑				^**^↑	^*^↑
Propionic acid	^***^↓	^***^↑	^***^↑			
BCFAs	^***^↓	^***^↑				
Ammonium	^***^↓	^***^↑	^*^↑		^**^↓	^*^↓

The groups are reported named as AB = antibiotic stimulation, CTR = control period, NT = non-treated microbiota, and TR = treated microbiota. Each column represents the comparison between groups, the asterisks represent the multiple comparisons of means (Tukey contrasts) *post-hoc* test significance (^***^ = 0 < *p*-value < 0.001, ^**^ = 0.01 < *p*-value < 0.01, ^*^ = 0.01 < *p*-value < 0.05), while the arrows tell whether the metabolite concentration increases (↑) or decreases (↓) in the comparison.

## 4 Discussion

The aim of this research was to develop an *in vitro* model to mimic a condition that reproduces in taxonomic and biodiversity terms the dysbiosis that occurs in dogs in real life following antibiotic administration, but also in cases of other intestinal issues such as episodes of diarrhea. Due to similarities between SHIME^®^ and SCIME™, some human experimental setups were adapted to meet the canine antibiotic-induced dysbiosis pattern. Although *in vivo* trials are the golden standard of studying disease processes, testing ingredients or even products for effectiveness, they are often too long and expensive (Nixon et al., 2019). In addition, despite their clinical importance, *in vivo* trials often do not succeed in unveiling how treatment mechanisms of action influence microbiota composition and functions (Duysburgh et al., 2021). Moreover, nowadays pet owners and consumers are very sensitive to the issue of animal testing and claims such as “Cruelty Free” or “Not Tested on Animals,” and international agencies as FDA (Food and Drug Administration) and EFSA (European Food Safety Authority) support the development and use of alternatives to whole-animal testing (FDA, 2022; EFSA, 2024). Likewise, European legislation for the protection of pets is very rigid and aims to reduce the use of dogs as laboratory animals, encouraging the development and validation of *in vitro* models instead (Council directive 2010/63/EU, 2010). In this context, the main objective of the current study was to investigate if the administration of a selected amount of broad-spectrum antibiotic to a healthy canine microbiota could trigger dysbiosis and induce changes on the level of the same markers that occur *in vivo*. This would establish if an *in vitro* model could help to prevent or reduce the use of dogs as laboratory animals.

Regarding the simulated microbial community composition, it was observed that samples taken after antibiotic administration were significantly different from those taken during the previous control week, which was considered a “healthy” microbiota condition (eubiosis). The results showed significant changes in microbial communities and activity, similar to data observed in fecal samples from dogs with acute diarrhea, which is often coupled with intestinal dysbiosis, such as decreased microbial richness, lower SCFAs production and altered microbial composition (Guard et al., 2015). In this regard, rarefaction curves and alpha diversity data showed that during antibiotic stimulation, the bacterial diversity in all vessels was significantly lower compared to the control week. Lower alpha diversity (Shannon and Chao1 index) is a marker of dysbiosis and gastrointestinal diseases (Félix et al., 2022) and this pattern is also seen *in vivo* when sequencing fecal sample of dogs with acute diarrhea compared to healthy ones (Suchodolski et al., 2012; Guard et al., 2015; Chaitman et al., 2020). In addition to the reduction of microbial richness, antibiotic administration also impacted the metabolic activity of canine microbiota, as observed *in vivo*. SCFAs are the major metabolic products of anaerobic fermentation by microbial communities that colonize the mammalian gut (Louis and Flint, 2017) and a reduction of SCFAs production is associated with dysbiosis and many canine disease processes (Suchodolski, 2016). During dysbiosis week, triggered by antibiotic treatment, a significant reduction of propionate production in all donors was observed, reproducing the same trend observed in fecal samples of dogs with acute diarrhea (Guard et al., 2015; Félix et al., 2022). Guard et al. speculated that the decreased fecal propionic acid could possibly be due to lower production and/or increased absorption into the gut epithelium during stages of acute diarrhea. In the current *in vitro* model, since the absorption is excluded, the decreased propionic acid can be correlated to the decrease of microbial richness due to antibiotic treatment. Propionate plays a key role in canine gut wellness (Minamoto et al., 2019) and many studies report a lower concentration of propionate in the feces of dogs with dysbiosis compared to healthy animals (Guard et al., 2015; Félix et al., 2022). The main propionate-producing bacteria belong to Bacteroidota and Bacillota (Negativicutes class), which produce propionate through the succinate pathway, from sugar fermentation (Louis and Flint, 2017). During dysbiosis week the reduced propionate production can be correlated with the decreased abundance phylum Bacteroidota and Bacillota and this result overlaps with the data observed *in vivo* (Bell et al., 2008; Suchodolski et al., 2012; Guard et al., 2015). Moreover, the decreased propionate synthesis may be due to the decreased abundance of bacteria belonging to Negativicutes, such as *Megamonas*, which was strongly impacted by antibiotic treatment in all donors. Guard et al. (2015) found out that, along the general reduction of SCFAs concentration, the proportion of butyric acid was significantly increased in fecal samples from dogs with acute diarrhea, compared to healthy dogs. In the current *in vitro* model, comparing control week and antibiotic week, the concentration of butyrate significantly decreased. For this fatty acid, a strong donor dependent variability was found, as seen in Supplementary Figure 1. In this regard, in donors 2, 3, 4, and 6, the production of butyrate increased, while it decreased in donor 1 and 5. Donors 2, 3, 4, and 6 had a typical canine microbial community, mainly composed by Bacillota, Fusobacteria, Bacteroidota, Pseudomonadota, and Actinomycetota (Pilla and Suchodolski, 2020). Conversely, in donors 1 and 5 the abundance of Fusobacteria was very low and the microbial community composition was more similar to human gut microbiota, where some species belonging to Fusobacteriaceae family are even correlated with colorectal cancer (Nawab et al., 2023). We can speculate that the difference in microbial communities, both in healthy dogs and in those with a dysbiosis condition, can be related to the individual dog's habits and surroundings. For instance, *Fusobacterium* abundance is increased in dogs spending time outdoors and it is also reported that pets and pet owners can share some taxa (Song et al., 2013). Conversely, the increased butyrate concentration may be due to the increased abundance of *Fusobacterium* spp., which can produce butyrate from peptide and amino acid fermentation through glutamate and lysine degradation pathways (Louis and Flint, 2017) and the phylum Fusobacteria was also increased in dogs with acute hemorrhagic diarrhea (Suchodolski et al., 2012). Obviously, these data have to be considered as preliminary since this study was performed using only six donors, and the results should be confirmed with a larger number of donors. Other microbiota metabolites, such as acetate, BCFAs and ammonia, tended to decrease during dysbiosis week. Their lower concentration can be generally related to lower Shannon and Chao1 index. It can be speculated that the lower alpha diversity mimics the reduction of bacteria during dysbiosis, because of the increased stool frequency (Vázquez-Baeza et al., 2016). The lower acetate production can be related to the decreased abundance of members of Bacillota (mainly *Megamonas*, but also *Fecalitalea* and *Phascolarctobacterium*) and Bacteroidota (*Alloprevotella* and *Prevotella* 9). The lower BCFAs concentration can be linked to the decreased abundance of *Bacteroides*. Moreover, during dysbiosis week a significant increase of Pseudomonadota, especially in donors with lower abundance of *Fusobacterium*, was observed. Pseudomonadota typically occur in small number in gut microbiota and fecal samples and their increase is often associated with dysbiosis and gastrointestinal diseases (Pilla and Suchodolski, 2020).

Having challenged the microbiota with antibiotic treatment, the current study investigated, as secondary outcome, whether a change in the SCIME™ feed preparation (supposed to reproduce a change in the diet) could have any effect in microbiota recovery. In this regard, the aim of this second part of the experiment was to evaluate if a higher amount of prebiotic ingredient in the nutritional medium could induce a better recovery of microbiota, since it is known that the microbiota may not fully recover after an episode of acute diarrhea (Chaitman et al., 2020). Some studies have shown the potential of prebiotic ingredients in limiting the destructive effect of antibiotic treatments on the intestinal microbiota by promoting faster recovery of gut homeostasis (Sanders et al., 2019). In this regard, a petfood with a higher amount of prebiotic ingredients was chosen in the second part of the experiment. It is generally assumed that dietary changes and complementary feeds are a more natural alternative to conventional pharmacological approach to intestinal issues, as it also happens for dermatological disorders (Marchegiani et al., 2020). It has been widely suggested that complementary feeds containing prebiotic, probiotic and symbiotic ingredients can modulate gut microbiota and could potentially prevent acute diarrhea in dogs at risk or shorten the duration of the dysbiosis (Mekonnen et al., 2020). In this context, pet owners are becoming increasingly aware of the quality and effectiveness of the dietetic formulas and complementary feeds on the market (ASSALCO, 2023).

One week after the end of antibiotic administration, alpha diversity index significantly increased compared to the previous control week, in both arms of the experiment. This behavior can be explained as recovery from the dysbiosis trigger: moreover, rarefaction curves plotting OTUs data (Figure 5) show that a greater number of species was detected in the treated arm than in non-treated arm. This positive trend can be due to the higher amount and variety of fibers included in the prebiotic-enriched petfood, compared to standard petfood (compositions showed in Table 2). In fact, reduced richness, common during acute dysbiosis, can facilitate the invasion of pathogens, that could colonize niches otherwise occupied by the endogenous microbiota (Britton and Young, 2012). The data obtained from treatment week indicated that the administration of both nutritional mediums improved the conditions in each colonic vessel, compared to the previous week. It was found that treatment negatively impacted ammonium production (compared to the CTR *p*-value = 0.00105 and the NTR: *p*-value = 0.02410): ammonia has been linked with proteolytic fermentation and is a potentially harmful microbiota metabolite, correlated to foul fecal odor and colon carcinogenesis (Lin and Visek, 1991; Félix et al., 2010). Decreased ammonia production can be correlated to the lower percentage of crude protein and the inclusion of *Yucca schidigera* in the prebiotic-enriched feed, since this plant is known to reduce fecal oudors and ammonia (Cheeke, 2000; Vierbaum et al., 2019).

In the two groups, no strong differences were observed. The little differences observed in standard feed and prebiotic-enriched feed can be explained by the little difference in their composition: a higher concentration of prebiotics or a longer duration of the treatment could have given different results. As seen in PCoA plots (Figure 6A) sample results overlap those from control week and microbial community analysis showed high inter-individual variation. During treatment week, the sample was taken one week after the end of antibiotic trigger. It can be speculated that, in this *in vitro* model recovery occurs quite quickly and more differences in microbial communities could be seen sampling more often (e.g., every day between the last day of the antibiotic trigger). In addition, this result can be explained by the similar composition of the two nutritional mediums: both commercial diets include prebiotic ingredients (they share dried beet pulp and dried brewer's yeast) and the relative abundance of commercial feed in the nutritional medium is low (0.9%). Regarding SCFAs, it was found that the acetate and propionate increase during the week after antibiotic stimulation, but there is no difference between groups, that were given either standard petfood or prebiotic-enriched petfood. Conversely, it was found that treatment positively impacted butyrate production compared to the CTR (*p*-value = 0.00115) and NTR (*p*-value = 0.01669). This can be explained by the higher amount of fibers in prebiotic-enriched feed. Butyrate is known to decrease the permeability of the intestinal epithelial lining by increasing the expression of tight junction proteins and reinforcing colonic defense barriers by increasing antimicrobial peptide levels and mucin production (Cook and Sellin, 1998; Wong et al., 2006; Antharam et al., 2013). It can be speculated that the increase in butyrate production may prevent over-growth of pathogens after an acute dysbisosis event.

As a limitation to this study, this *in vitro* work did not include a parallel *in vivo* validation, as happened for the validation of the SCIME™ model (Duysburgh et al., 2020). An additional *in vivo* validation would be favorable. Recent studies, such as the work of Argentini et al. (2022), confirm the rational of using *in vitro* models to reproduce microbial changes that would occur *in vivo* following antibiotic use.

Another limitation of the current work is that only a small number of animals were enrolled, partly due to cost and time restrictions. Also, all dogs, while all living in Ascoli Piceno (Italy), were on different diets and housed in different environments that were not controlled. Differing environments may influence intestinal microbiota. A larger number of enrolled donors would give more insights about microbiota modulation, due to the physiological interindividual variability in microbial communities. Anyway, we decided to select 6 donors for the study, based on literature research and previous publications where SCIME™ (Duysburgh et al., 2020; Verstrepen et al., 2021) and SHIME^®^ (Deyaert et al., 2023; Duysburgh et al., 2024) have been used. The SCIME™ model, as well as the SHIME^®^ and other chemostat models, allows the creation of an environment with highly reproducible and physiological conditions for the intestinal microbiota, by the means of a fecal inoculation of the system. In our case, by inoculating the system with the fecal material of 6 donors and considering only the distal colon (since we were interested in the effect of the antibiotic on the terminal part of the GI tract) we were able to introduce another variable in the system (prebiotic-enriched petfood compared to standard petfood). This because we had two replicates of the distal colon for each donor. This study can be considered a preliminary test and future experiments involving a larger sample size are needed to confirm or confute the results.

Another limitation of the study was that only a single antibiotic was used and it was known to cause dysbiosis. As stated in the abstract and introduction, the primary outcome of the study was to evaluate if SCIME™ could be used to mimic intestinal dysbiosis, as previously done employing SHIME^®^. For this reason, an already known trigger of dysbiosis *in vivo* was selected. In particular, fewer antibiotic are available as veterinary drugs for dogs, compared to those for human, and amoxicillin-clavulanic acid is one of them (Synulox, Clavobay, and Clavaseptin). Since information about therapeutical dosage and ADME are necessary to calculate the dose to administer to SCIME™, it was mandatory to select an antibiotic approved for dogs. Moreover, we decided to use only one antibiotic as a trigger after studying the latest papers (El Hage et al., 2019; Duysburgh et al., 2021). In addition, in a recent work from El Hage et al. a similar experimental setup (one antibiotic, six donors) was used (El Hage et al., 2019).

As the current study mainly focused on the validation of an acute dysbiosis model, especially in the distal colon region, it could be interesting to extend the setup to the conventional SCIME™ reactor, including proximal colon. Moreover, to further understand the effect of dysbiosis on canine microbiota, the inclusion of mucosal compartment could be useful (Verstrepen et al., 2021).

## 5 Conclusions

In conclusion, a dynamic *in vitro* model simulating canine antibiotic-induced dysbiosis was developed, with a focus on the distal colon-associated microbial community and its metabolites. The current study discovered that it is possible to mimic *in vitro* a condition that reproduces in taxonomic and biodiversity terms the dysbiosis that occurs in dogs in real life following antibiotic administration (whether it causes diarrhea or not) and during other conditions where, regardless of whether or not dogs received antibiotics, episodes of diarrhea occur (whether due to gastroenteritis, functional gastrointestinal disorders or IBD). Moreover, this new SCIME™ setup facilitated the reproduction of microbial and metabolic changes seen *in vivo* in fecal samples obtained from dogs with acute diarrhea, such as lower microbial diversity and decreased concentration of propionate.

The main goal of this work is that, upon inducing dysbiosis with antibiotic administration, the simulated canine microbiota reproduced the same patterns seen *in vivo* in cases of antibiotic-induced dysbiosis, indicating an interesting application potential in research related to canine gastrointestinal health and petfood development, and preventing the use of *in vivo* testing.

## Data Availability

The original contributions presented in the study are publicly available. This data can be found here: https://doi.org/10.6084/m9.figshare.26863042.v1.
