# Supplementary material for: Antibiotic-induced dysbiosis in the SCIME™ recapitulates microbial community diversity and metabolites modulation of in vivo disease
Source: Front Microbiol. 2024 Sep 12;15:1455839. doi: 10.3389/fmicb.2024.1455839 (PMC11424444; doi:10.3389/fmicb.2024.1455839)
Supplement: Supplementary file 1 [file Table_1.DOCX]

**Supplementary Material**

**Table S1** Canine donors.

| **Breed** | **Age** | **Experiment run** | **Name** |
| --- | --- | --- | --- |
| Toy Poodle | Adult | 1 | D1 |
| Springer Spaniel | Adult | 1 | D2 |
| Cane Corso | Mature | 2 | D3 |
| Mixed-breed | Mature | 2 | D4 |
| Jack Russell | Adult | 3 | D5 |
| Labrador Retriever | Adult | 3 | D6 |

**Table S2** Mean ± standard deviation of alpha diversity in different metrics (OTU, Chao1 index, Shannon index), divided by group (AB = antibiotic stimulation, CTR = control period, NT = non-treated microbiota, TR = treated microbiota).

|  | **AB** | **CTR** | **NT** | **TR** |
| --- | --- | --- | --- | --- |
| **OTU (mean ± sd)** | 48,8±12,30 | 55,25±11,50 | 55,10±14,42 | 50,93±9,93 |
| **Chao1 (mean ± sd)** | 48,95±12,36 | 55,46±11,68 | 55,43±14,79 | 51,19±10,33 |
| **Shannon (mean ± sd)** | 2,90±0,75 | 3,64±0,46 | 3,62±0,48 | 3,44±0,49 |

**Figure S3** Canine microbial metabolic activity in the SCIME™. The short chain fatty acids (SCFA), acetic acid (A), butyric acid (B) propionic acid (C) and branched acids boxplots for 6 donors tested. The groups are reported named as AB = antibiotic stimulation, CTR = control period, NT = non-treated microbiota, TR = treated microbiota.

**Figure S4** Canine microbial metabolic activity in the SCIME™. Ammonium boxplots for 6 donors tested. The groups are reported labelled as AB = antibiotic stimulation, CTR = control period, NT = non-treated microbiota, TR = treated microbiota.
