# Supplementary figures and images for: Antibiotic-induced dysbiosis in the SCIME™ recapitulates microbial community diversity and metabolites modulation of in vivo disease
Source: Front Microbiol. 2024 Sep 12;15:1455839. doi: 10.3389/fmicb.2024.1455839 (PMC11424444; doi:10.3389/fmicb.2024.1455839)

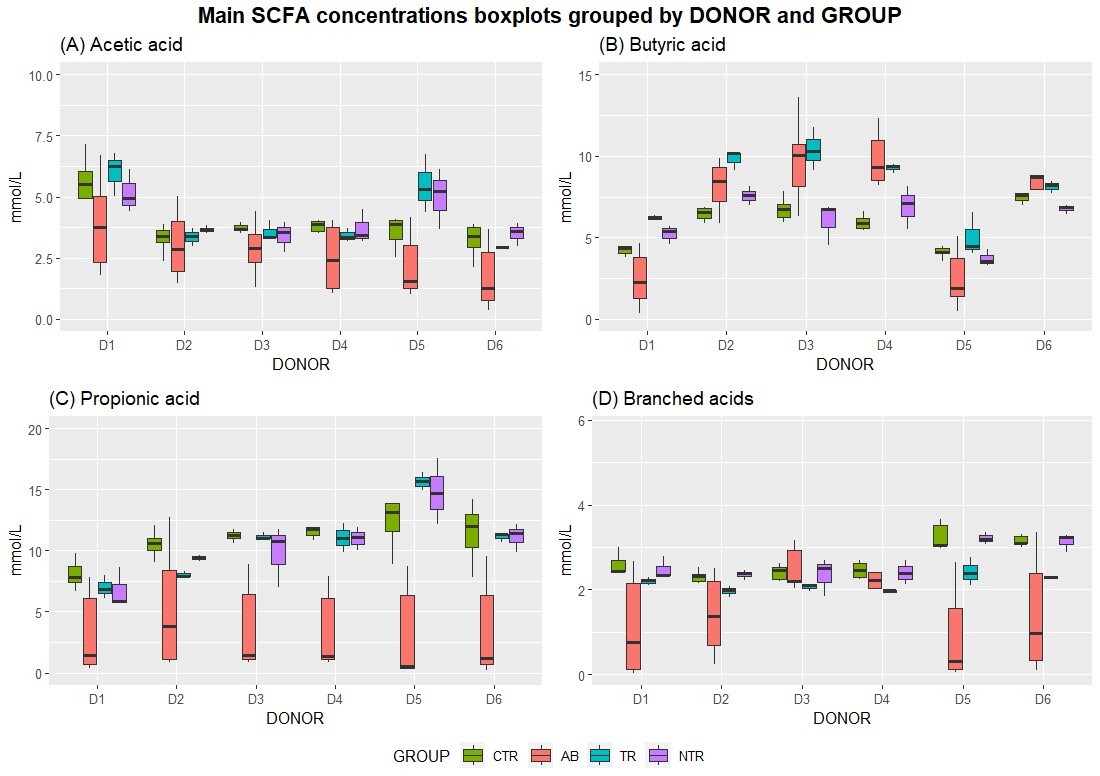

Supplement: Supplementary file 2 [file Image_1.JPEG]

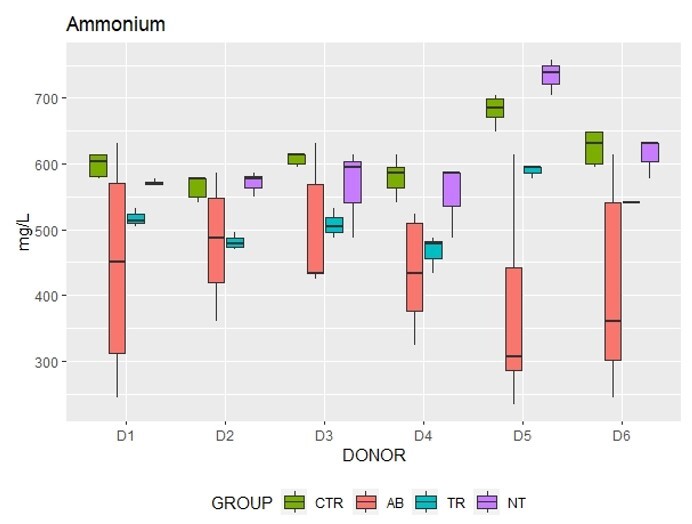

Supplement: Supplementary file 3 [file Image_2.JPEG]
